# Supplementary material for: SurGrID: controllable surgical simulation via Scene Graph to Image Diffusion
Source: Int J Comput Assist Radiol Surg. 2025 May 21;20(7):1421–9. doi: 10.1007/s11548-025-03397-y (PMC12226606; doi:10.1007/s11548-025-03397-y)
Supplement: Supplementary file 1 — (pdf 2652 KB) [file 11548_2025_3397_MOESM1_ESM.pdf]

Appendix A Scene Graph Generation

We assume our dataset $\mathcal{D}$ consists of (image, mask) pairs $(x_i, m_i) \in \mathcal{D}$. From each mask m_i, we build a Scene Graph $\mathcal{G}_i$ by extracting the masks' connected components. We then compute the centroid of each component. Each node of the final graph represents an individual connected component and encodes the component's d-*dim* class vector, the 2-*dim* spatial spreading of the component, and the component's 2-*dim* centroid coordinates. By explicitly encoding these values in the node features of the graph, we can precisely control the image synthesisation. In the graph's edges, we encode the spatial relationship of nodes, for which we check if nodes' components are touching spatially. Therefore, a SG for sample i is defined by $\mathcal{G}_i = (\mathcal{V}_i, \mathcal{E}_i)$ where $\mathcal{V}_i \in \mathbb{R}^{n \times (d+4)}$ are the node features, with n being the number of nodes in the graph, and $\mathcal{E}_i$ is the set of undirected edges between connected nodes. Our surgical SG $\mathcal{G}_i$ yields a compact yet spatially and semantically accurate representation of a surgical image with a clear interpretation, as presented in Figure A1. Further, graph representations allow seamless modification of generative model conditioning by changing node features, which we demonstrate in Section 4.

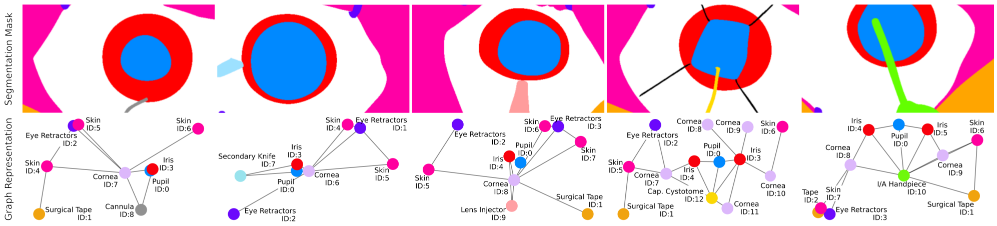

Fig. A1 Surgical Scene Graphs. Each connected component in the segmentation mask is assigned a node. We store the component's class label, spatial spreading, and centroid coordinate within the node features. Visualized in the bottom row, the node positions correspond to these centroid coordinates. Edges between nodes indicate that components are spatially connected in the segmentation mask.

Appendix B Pre-Training Embedding Space Visualisation

Figure B2 presents t-SNE visualizations of image and graph embeddings after the pre-training stage. The t-SNE plots in the first column are colour-coded by video ID, indicating that frames from the same video share the same colour. In contrast, the t-SNE plots in the second column are classified by the combinations of present tools.

These visualizations strongly support our claims. In the first column, when image features are classified by video ID, evident signs of clusterings can be seen, demonstrating that frames from the same video are grouped in the image embedding space. This clustering occurs regardless of the tools present in the frames, majorly due to shared anatomical features across frames. However, the top-right t-SNE plot shows little to no clustering when the embeddings are classified by tool combinations, reinforcing the

observation that image embeddings fail to encode tool-specific information effectively. With pre-trained graph embeddings, clear clusters emerge, as shown in the bottom-right t-SNE plot. This indicates that frames with similar surgical tools and positions are close in the embedding space, irrespective of their video origin. On the other hand, when graph embeddings are classified by video ID, there is minimal clustering, as shown in the bottom-left t-SNE plot. Overall, this demonstrates that our pre-training method captures embeddings that are not dominated by video-specific information and clusters frames with similar surgical tools and positions in the embedding space.

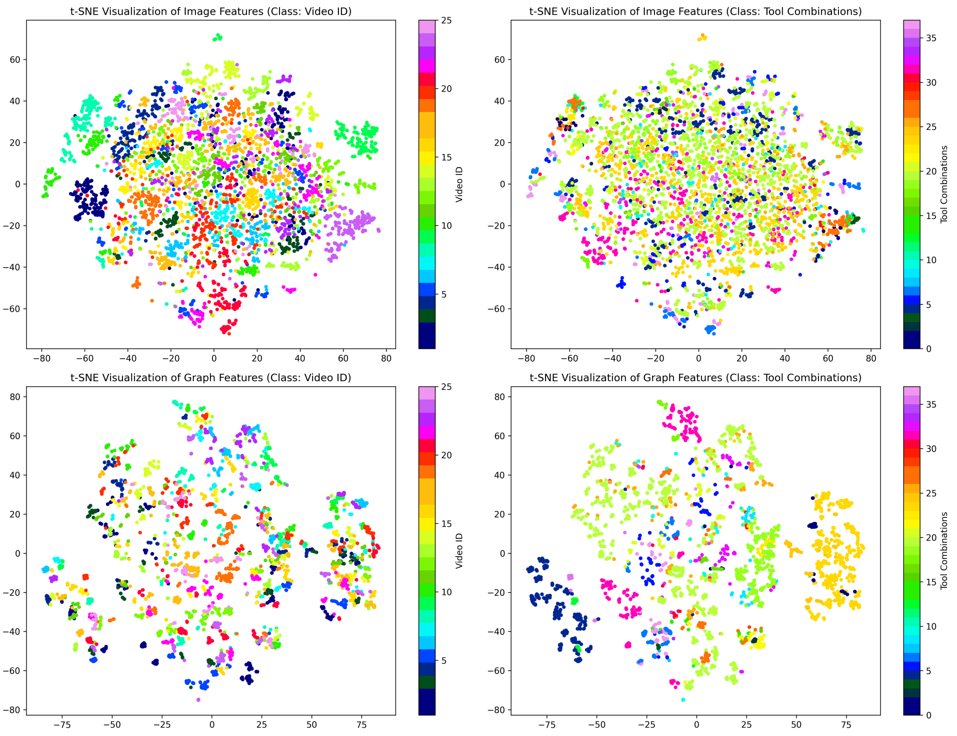

Fig. B2 **t-SNE Embedding Space Visualisation.**

Appendix C Expert Assessment Study - Feedback

After participation, we asked participants to explain their reasoning for assigning low or high values to the realism and coherence of the generated images. In summary, they mentioned that extreme changes to the input SG could be better reflected in the generated image. This primarily concerns the spatial positions of tools that are far from what the training dataset contains. The participants occasionally observed image parts that could be more realistic, e.g., off-looking anatomical textures, warped instruments, or a blurry or unrealistic interaction between tool and tissue. Additionally,

they observed inconsistencies within the mini-batch of generated images. Individual images would sometimes show wrong or no tools. Lastly, they also gave feedback on the graph representation, which they found easy to operate to condition the simulation. However, they wished for even more control, e.g., controlling the size of the pupil or the angle of the tools, and an even further simplified representation, e.g., not showing one node per component but rather per anatomy class. Nonetheless, we received overall positive feedback for the general idea and the generated images' realism and coherence with the conditioning, reflected in the scores in Section 4.5.

Appendix D Expert Assessment Study - GUI

This section presents the GUI used during our user study in Figure D3.

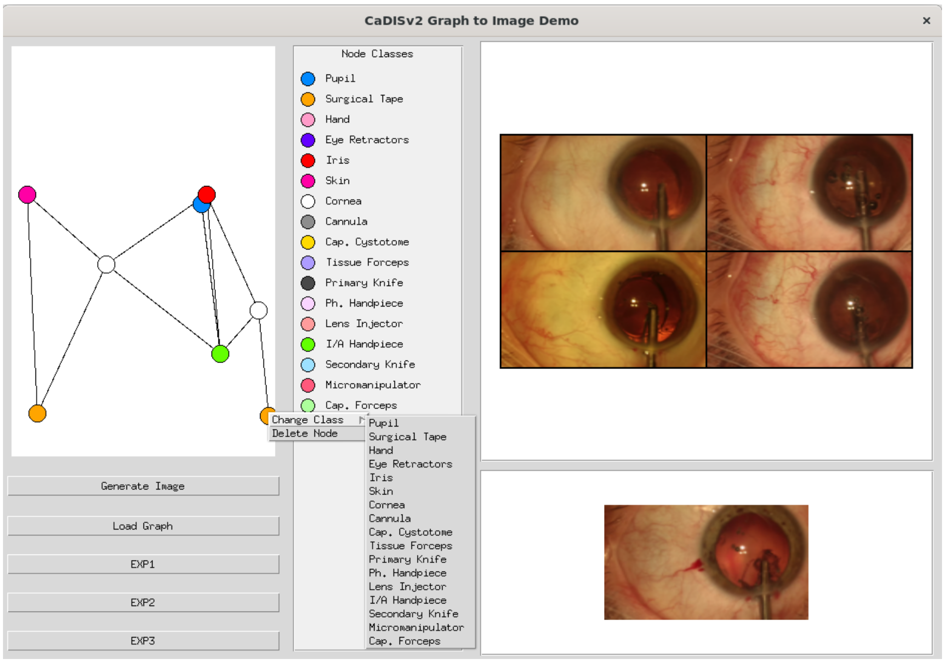

Fig. D3 Graphical User Interface for Expert Assessment. In our GUI, participants can load ground truth (image, graph) pairs (bottom right and left). The GUI allows the following operations for modifying the graph: Nodes can be *moved* spatially by drag-and-drop. They can be *deleted* or *changed into different classes* by right-clicking them. Further, *new nodes can be added* by right-clicking the empty space in the SG canvas. Eventually, participants can generate a mini-batch of four images using the currently displayed SG (top right).

Appendix E Limitations & Future Work

In future work, we will primarily address the feedback from our user study, hence exploring ways to improve the usability of the GUI and SG representations. We also

want to allow additional control over the pupil size and the tool angle. Since we see frame-based simulation only as a first step in building a surgical simulator, we will further explore sequence generation in the future. For example, sequential editing of an initial reference frame could substantially improve the temporal consistency of image information not encoded in the SG representation, such as textures or blood vessels. Additionally, we will explore other surgical domains in future research. We expect these directions to strengthen the possibilities for clinical translation significantly.
